# Butyrate in combination with forskolin alleviates necrotic enteritis, increases feed efficiency, and improves carcass composition of broilers

**DOI:** 10.1186/s40104-021-00663-2

**Published:** 2022-02-10

**Authors:** Qing Yang, Binlong Chen, Kelsy Robinson, Thiago Belem, Wentao Lyu, Zhuo Deng, Ranjith Ramanathan, Guolong Zhang

**Affiliations:** 1 Department of Animal and Food Sciences, Oklahoma State University, Stillwater, OK USA; 2 College of Animal Science, Xichang University, Xichang, Sichuan China; 3 Present address: Poultry Production and Product Safety Research Unit, USDA–Agricultural Research Service (ARS), Fayetteville, AR USA; 4 State Key Laboratory for Managing Biotic and Chemical Threats to the Quality and Safety of Agro-products, Institute of Agro-product Safety and Nutrition, Zhejiang Academy of Agricultural Sciences, Hangzhou, China; 5 Center for Excellence in Hip Disorders, Texas Scottish Rite Hospital for Children, Dallas, TX USA

**Keywords:** Antibiotic alternatives, Butyrate, Forskolin, Necrotic enteritis, Poultry

## Abstract

**Background:**

The emergence of antimicrobial resistance has necessitated the development of effective alternatives to antibiotics for livestock and poultry production. This study investigated a possible synergy between butyrate and forskolin (a natural labdane diterpene) in enhancing innate host defense, barrier function, disease resistance, growth performance, and meat quality of broilers.

**Methods:**

The expressions of representative genes involved in host defense (*AvBD9* and *AvBD10*), barrier function (*MUC2*, *CLDN1*, and *TJP1*), and inflammation (*IL-1β*) were measured in chicken HD11 macrophages in response to butyrate and forskolin in the presence or absence of bacterial lipopolysaccharides (LPS). Intestinal lesions and the *Clostridium perfringens* titers were also assessed in *C. perfringens-*challenged chickens fed butyrate and forskolin-containing *Coleus forskohlii* (CF) extract individually or in combination. Furthermore, growth performance and carcass characteristics were evaluated in broilers supplemented with butyrate and the CF extract for 42 d.

**Results:**

Butyrate and forskolin synergistically induced the expressions of *AvBD9*, *AvBD10*, and *MUC2* in chicken HD11 cells (*P* < 0.05) and the synergy was maintained in the presence of LPS. Butyrate and forskolin also suppressed LPS-induced *IL-1β* gene expression in HD11 cells in a synergistic manner (*P* < 0.05). The two compounds significantly reduced the intestinal lesions of *C. perfringens-*challenged chickens when combined (*P* < 0.05), but not individually. Furthermore, butyrate in combination with forskolin-containing CF extract had no influence on weight gain, but significantly reduced feed intake (*P* < 0.05) with a strong tendency to improve feed efficiency (*P* = 0.07) in a 42-d feeding trial. Desirably, the butyrate/forskolin combination significantly decreased abdominal fat deposition (*P* = 0.01) with no impact on the carcass yield, breast meat color, drip loss, or pH of d-42 broilers.

**Conclusions:**

Butyrate and forskolin has potential to be developed as novel antibiotic alternatives to improve disease resistance, feed efficiency, and carcass composition of broilers.

**Supplementary Information:**

The online version contains supplementary material available at 10.1186/s40104-021-00663-2.

## Introduction

A growing number of countries have phased out the use of antibiotics for growth promotion and disease prevention in livestock animals due to a growing concern over antibiotic resistance [1]. Consequently, alternatives to antibiotics are imperative to maintain the health and production efficiency of livestock. Enhancing animal innate immunity through stimulation of endogenous host defense peptide (HDP) synthesis has emerged as a novel alternative approach to antibiotics [2, 3]. HDPs, also known as antimicrobial peptides, are ubiquitously expressed in phagocytes and epithelial cells lining the digestive, respiratory, urinary, and reproductive tracts, and constitute an important component of host immune defense [4–6]. Defensins and cathelicidins are 2 major families of HDPs in vertebrates, and 14 β-defensins (AvBD1–14) and 4 cathelicidins (CATH1–3 and CATHB1) have been reported in chickens [5]. Besides the ability to directly kill a broad spectrum of microbes through membrane disruption, HDPs possess a variety of immunomodulatory activities such as chemotaxis, endotoxin neutralization, suppression of inflammation, and activation of adaptive immunity [6]. Because of membrane-lytic antimicrobial and immune regulatory activities, HDPs are less likely to trigger microbial resistance than conventional antibiotics [7].

Insufficient HDP production has been implicated in increased susceptibility to infectious diseases, while enhancing endogenous HDP synthesis is capable of strengthening host immunity and conferring protection [6, 8]. Besides infection and inflammation, HDP synthesis can be modulated by a variety of small-molecule compounds such as histone deacetylase inhibitors, fatty acids, and phytochemicals [3, 9, 10]. For example, butyrate, a major short-chain fatty acid (SCFA) produced by intestinal bacterial fermentation of undigested dietary carbohydrates, is essential to intestinal health and homeostasis by regulating energy metabolism, inflammation, immunity, and barrier integrity of the intestinal epithelial cells [11, 12]. Recently, butyrate was found to be a potent HDP inducer in humans and several other animal species [3]. Most of the butyrate functions including its HDP-inducing activity are mediated through inhibiting histone deacetylases or interacting with G-protein coupled receptors such as GPR41, GPR43, and GPR109A [11].

Forskolin (FSK), a labdane diterpene isolated from the roots of an Indian plant *Coleus forskohlii* (CF), is also capable of stimulating HDP synthesis in humans [13] and chickens [14]. FSK modulates a variety of physiological functions such as promoting lipolysis and thermogenesis, appetite regulation, and anti-inflammation by acting as a direct agonist of adenylyl cyclase, which in turn activates cyclic adenosine monophosphate (cAMP) signaling to influence gene transcription [15, 16].

We previously showed a strong synergy between butyrate and FSK in inducing *AvBD9* gene expression in chickens [14]. However, the synergy between butyrate and FSK in barrier integrity and disease resistance has remained unknown. Therefore, this study was aimed at investigating the role of butyrate and FSK in modulating innate immunity, inflammation, and barrier function. We also studied the efficacy of the two natural compounds in the resistance of necrotic enteritis (NE) in broiler chickens, which is among the most economically significant diseases in chickens caused by a Gram-positive bacterium, *Clostridium perfringens* [17]. The impact of the two compounds on growth performance, carcass traits, and meat quality was also evaluated to further explore their potential as novel alternatives to antibiotics for use in broilers.

## Materials and methods

### Cell culture and treatments

Chicken HD11 macrophage cells [14, 18] were cultured in complete RPMI 1640 (HyClone, Logan, UT, USA) containing 10% fetal bovine serum (Atlanta Biologicals, Flowery Branch, GA, USA), 100 U/mL penicillin, and 100 μg/mL streptomycin (Lonza, Walkersville, MD, USA). After seeding at 1 × 10^6^ cells/well overnight in 12-well cell culture plates, cells were treated in duplicate with 2 mmol/L sodium butyrate (Sigma, St. Louis, MO, USA) or 5 μmol/L FSK (Santa Cruz Biotechnology, Dallas, TX, USA) individually or in combination. After 24-h incubation at 37 °C and 5% CO_2_, cells were stimulated with 10 ng/mL lipopolysaccharide (LPS) (*E. coli* O55:B5, Sigma-Aldrich) for another 3 h, followed by total RNA isolation, reverse transcription, and quantitative PCR (qPCR) analysis of the expressions of various genes as described below. Three independent experiments were conducted to ensure the reproducibility of the results.

### Gene expressions analysis

Total RNA was isolated using RNAzol RT (Molecular Research Center, Cincinnati, OH, USA) and quantified using Nanodrop, followed by quantitative reverse transcription PCR (RT-qPCR) analysis of the expression levels of *AvBD9*, *AvBD10*, mucin 2 (*MUC2*), claudin 1 (*CLDN1*), tight junction protein 1 (*TJP1*), interleukin 1β (*IL-1β*), and glyceraldehyde 3-phosphate dehydrogenase (*GAPDH*) as described [14] using iTaq Universal SYBR Green Supermix (Bio-Rad, Hercules, CA, USA) and gene-specific primers (Table 1) on a CFX96™ Real-Time PCR Detection System (Bio-Rad). PCR program was an initial activation at 95 °C for 30 s, followed by 40 cycles at 94 °C for 5 s, 60 °C for 30 s and 94 °C for 5 s. Relative fold changes of gene expression levels were calculated using the 2^-ΔΔCt^ method normalized against *GAPDH*.

**Table 1:** The primer sequences used in RT-qPCR

Gene	Primer sequences (5′ → 3′)	Product size, bp	GenBank accession number
*AvBD9*	Forward: GCAAAGGCTATTCCACAGCAG	211	NM_001001611.2
	Reverse: AGCATTTCAGCTTCCCACCAC		
*AvBD10*	Forward: TGGGGCACGCAGTCCACAAC	298	NM_001001609.2
	Reverse: ATCAGCTCCTCAAGGCAGTG		
*MUC2*	Forward: TCTGGAGAGAGTTGTCCTGAC	105	JX284122.1
	Reverse: TCCTTGCAGCAGGAACAACT		
*CLDN1*	Forward: TTCCAACCAGGCTTTATGATG	140	NM_001013611.2
	Reverse: TGCAGAGTCAGGTCAAACAGA		
*TJP1*	Forward: CATCAGCCAGAAGAGAACCAG	117	XM_037393868.1
	Reverse: CCAAGAACAAAAGTGGTATGC		
*IL-1β*	Forward: GACATCTTCGACATCAACCAG	215	XM_015297469.1
	Reverse: CCGCTCATCACACACGACAT		
*GAPDH*	Forward: GCACGCCATCACTATCTTCC	356	NM_204305.1
	Reverse: CATCCACCGTCTTCTGTGTG		

### Chicken model of NE

All animal procedures were approved by the Institutional Animal Care and Use Committee of Oklahoma State University under protocol number AG-16-10. A total of 162 newly-hatched male Cobb broilers were obtained from a commercial hatchery (Cobb-Vantress Hatchery, Siloam Springs, AR, USA) and reared in an environmentally controlled room under standard management as recommended by Cobb-Vantress. Chickens were randomly assigned to 1 of 6 treatments in 18 floor pens with fresh pinewood shavings, 9 birds/pen, and 3 pens/treatment. Animals were provided ad libitum with tap water and a non-medicated commercial crumble corn-soybean diet (DuMOR Chick Starter/Grower 20%, Tractor Supply Co., Brentwood, TN, USA). At d 10, broilers in 12 pens were fed diets supplemented with microencapsulated butyrate (1 g/kg diet, CM3000® containing 30% pure sodium butyrate, King Techina, Hangzhou, China), 20% FSK-containing CF extract (10 mg/kg diet, PureBulk, Roseburg, Oregon, USA), or a combination of sodium butyrate (1 g/kg diet) and CF extract (5 or 10 mg/kg), respectively, while the remaining chickens in 6 pens continued to access the basal diet.

After overnight fasting at d 13, 3 chickens were randomly selected from each floor pen, weighed, and transferred to 18 battery cages with 3 animals/cage and three cages/treatment. Chickens in 5 treatments were subjected to daily challenge with an overnight culture of *C. perfringens* (approximately 4–5 × 10^8^ CFU/mL) mixed 1:1 (v/w) with 100 g of respective diets/cage for 4 consecutive days from d 14 to 17, while chickens in the mock-infected control group were fed the basal diet mixed 1:1 (v/w) with sterile fluid thioglycollate broth as described [19]. A *netB-* and *tpeL-*positive *C. perfringens* strain Brenda B (kindly provided by Dr. Lisa Bielke at the Ohio State University, Columbus, OH) [20] was sequentially passaged in chopped cooked meat medium and fluid thioglycollate medium prior to inoculation of chickens. On d 18, chickens were weighed individually and euthanized by CO_2_ asphyxiation prior to sample collection. Gross lesions of NE in the duodenum and jejunum of each chicken were graded separately in a blind manner using a 0–6 scoring system as described [17]. Moreover, a mid-jejunal segment and the digesta from the distal jejunum and cecum were collected from each animal and stored at − 80 °C until further analysis.

### Quantification of intestinal *C. perfringens*

The jejunal and cecal *C. perfringens* were quantified using a standard curve-based qPCR method as described [21]. Briefly, bacterial genomic DNA from pure *C. perfringens* culture or intestinal digesta samples was extracted using the ZR Fecal DNA MicroPrep Kit (Zymo Research, Irvine, CA) and quantified using Nanodrop. *C. perfringens* was amplified using the primers 5′-AAAGATGGCATCATCATTCAAC-3′ (forward) and 5′-TACCGTCATTATCTTCCCCAAA-3′ (reverse) [22] on a CFX96™ Real-Time PCR Detection System (Bio-Rad) with an initial activation at 95 °C for 30 s, followed by 40 cycles at 94 °C for 5 s, 60 °C for 30 s and 94 °C for 5 s. The *C. perfringens* titer in each digesta sample was calculated and expressed as log_10_ CFU/g digesta based on the standard curve developed using 10-fold serial dilutions of *C. perfringens* genomic DNA.

### Growth performance of broilers

Two separate feeding trials were conducted to evaluate the effect of butyrate and FSK on the growth performance of healthy broilers reared under standard management. In the 21-d trial, a total of 288 newly-hatched male Cobb chicks were randomly assigned to 1 of 6 treatments with 6 floor pens/treatment and 8 animals/pen. Six dietary treatments were a non-medicated commercial basal crumble diet (DuMOR Chick Starter/Grower 20%, Tractor Supply Co.) supplemented with or without microencapsulated sodium butyrate (1 g/kg diet; CM3000®, King Techina), 20% FSK-containing CF extract (25 mg/kg, PureBulk), or a combination of microencapsulated sodium butyrate (1 g/kg) and CF extract (5, 10, or 25 mg/kg), respectively. Body weight (BW) gain and feed intake were recorded weekly for 3 weeks by pen (*n* = 6). Average daily gain (ADG), average daily feed intake (ADFI), and feed conversion ratio (FCR) were calculated and compared among all treatments.

In the 42-d feeding trial, a total of 288 day-of-hatch male Cobb broiler chicks were randomly distributed into 4 treatments with 9 floor pens/treatment and 8 animals/pen. Birds were given a non-medicated standard corn-soybean basal diet or the basal diet supplemented with 1 g/kg microencapsulated sodium butyrate and 10 mg/kg CF extract individually or in combination. Broilers were raised on 3-phase diets (starter, grower, and finisher) that were changed every 2 weeks. The starter diet (21.5% crude protein) was mash, while grower (20% crude protein) and finisher diets (18.0% crude protein) were pelleted. Chickens were weighed weekly for 6 weeks by pen for the calculation of ADG, ADFI, and FCR (*n* = 9).

### Carcass traits and meat quality

In the 42-d feed trial described above, 3 broilers from each pen were randomly selected for the analysis of carcass characteristics and breast meat quality on d 28 (*n* = 27). On d 42, all 5 remaining birds from each pen were weighed and euthanatized for carcass trait and meat quality measurements (*n* = 45). Broilers were euthanized by CO_2_ asphyxiation after 12-h feed withdrawal, followed by ventral neck cutting, bleeding, de-feathering, and evisceration. The carcass yield was calculated as the percentage of eviscerated carcass weight, relative to live weight. Moreover, both left and right sides of the breast muscle (pectoral major and minor) were removed and weighed. The abdominal fat pad was collected and weighed as described [15]. The yields of the breast meat and abdominal fat were calculated as percentages of eviscerated carcass weight.

The breast muscle was weighed after placing it in a plastic bag at 4 °C for 24 h to estimate the drip loss, which was expressed as the percentage of initial breast muscle weight as described [16]. The color of the right pectoral major muscle was determined on each sample in duplicate with 1 reading in the anterior and the other in the posterior portion of the muscle using MiniScan XE Plus Spectrophotometer (2.5 cm aperture, Illuminant A, and 10° standard observer angle; HunterLab Associates, Reston, VA) and the CIE system (L* = lightness; a* = redness; b* = yellowness). All readings were taken on the skin side surface in an area free of obvious color defects (over scald, bruises, and blood accumulation) [23]. The right side of the pectoral minor muscle was used to determine pH after chilling at 4 °C for 24 h in self-sealed plastic bags. Values of pH were collected at 3 different places of each sample with portable meat pH meter (Model HI99163, Hanna Instruments, Woonsocket, Rhode Island) equipped with an insertion glass electrode calibrated in buffers at pH 4.00 and 7.00 at ambient temperature [16].

### Statistical analysis

Data analysis and visualization were performed using Prism (GraphPad Software Inc., La Jolla, CA) or SPSS 23.0 (IBM, Chicago, IL). Results were expressed as means ± standard errors of the mean (SEM), and significance was determined using one-way ANOVA and post hoc Tukey’s test. Statistical significance was considered at *P* ≤ 0.05, while tendency was considered at 0.05 < *P* ≤ 0.10

## Results

### Regulation of HDP, barrier function, and inflammatory cytokine gene expression in HD11 cells by butyrate and FSK

**Fig. 1:**
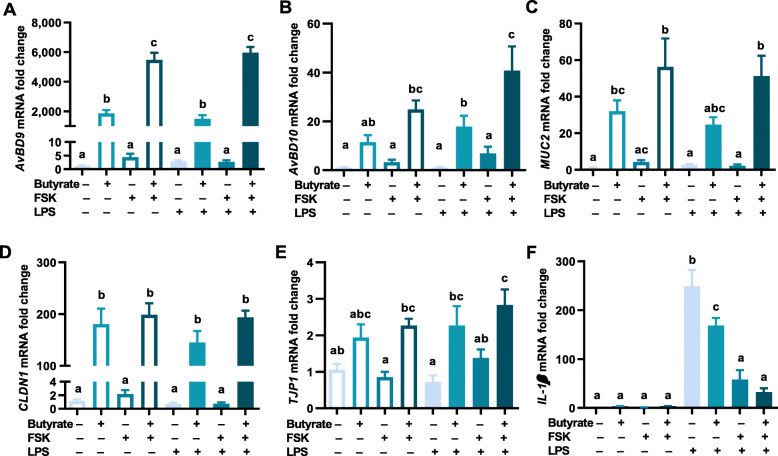
Regulation of host defense peptide, barrier function, and inflammatory cytokine gene expressions in macrophages by butyrate and forskolin (FSK). Chicken HD11 macrophage cells were treated in duplicate with 2 mmol/L sodium butyrate and 5 μmol/L FSK separately or in combination for 24 h, followed by stimulation with 10 ng/mL lipopolysaccharides (LPS) for another 3 h. Quantitative reverse transcription PCR (RT-qPCR) was performed to measure the expressions of avian β-defensin 9 (*AvBD9*) (**A**), *AvBD10* (**B**), mucin 2 (*MUC2*) (**C**), claudin 1 (*CLDN1*) (**D**), tight junction protein 1 (*TJP1*) (**E**), and interleukin 1β (*IL-1β*) (**F**). Results were presented as means ± SEM of 3 independent experiments. Statistical significance (*P* < 0.05), denoted by uncommon superscripts, was determined using one-way ANOVA and post hoc Tukey’s test

To explore a possible synergy between butyrate and FSK in modulating major genes involved in innate defense, barrier function, and inflammation, chicken HD11 macrophage cells were treated with sodium butyrate and FSK individually or in combination for 24 h, followed by stimulation with LPS for another 3 h. The mRNA expression levels of representative HDP, barrier function, and inflammatory cytokine genes were evaluated using RT-qPCR. While butyrate and FSK induced *AvBD9* gene expression separately, a combination of butyrate and FSK showed an obvious synergy, giving an additional 3-fold increase in *AvBD9* expression over butyrate alone (*P* < 0.05) (Fig. 1A), consistent with our earlier report [14]. Moreover, *AvBD9* induction was maintained in the context of LPS stimulation. Similarly, butyrate synergized with FSK in enhancing the expressions of *AvBD10* (Fig. 1B) and *MUC2* (Fig. 1C), regardless of the LPS challenge. Additionally, butyrate augmented the expressions of *CLDN1* and *TJP1* in HD11 cells, while FSK was largely ineffective (Fig. 1D and E). The butyrate/FSK combination showed no obvious synergy in *CLDN1* and *TJP1* expressions in the presence or absence of LPS (Fig. 1D and E). Furthermore, butyrate, FSK, or the combination had little effect on the expression of *IL-1β* in HD11 cells, indicating that the 2 compounds are not proinflammatory (Fig. 1F). Desirably, butyrate and FSK separately suppressed LPS-induced *IL-1β* expression (*P* < 0.05), and such a suppression became more pronounced in response to the butyrate/FSK combination (Fig. 1F). Taken together, butyrate synergized with FSK to suppress inflammation, while enhancing the expressions of HDP and *MUC2* genes, with no obvious synergy in regulating tight junction genes.

### Alleviation of NE in chickens by butyrate and FSK-containing plant extract

**Fig. 2:**
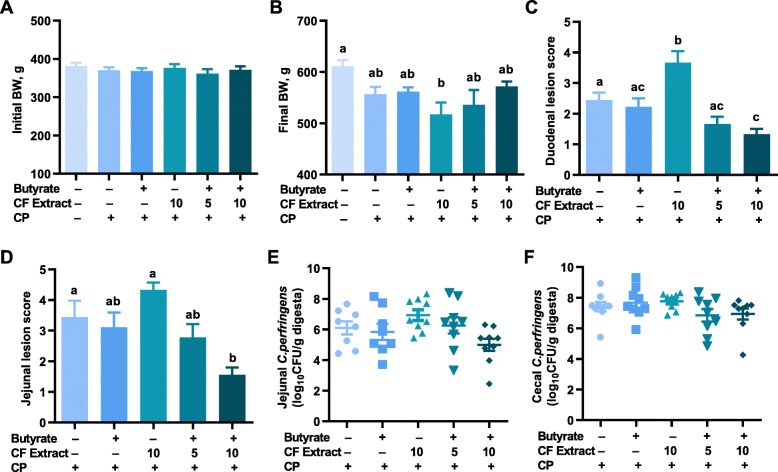
Alleviation of necrotic enteritis by butyrate and forskolin (FSK). Day-10 male Cobb broilers were supplemented with or without microencapsulated sodium butyrate (1 g/kg diet), FSK-containing *Coleus forskohlii* (CF) extract (10 mg/kg) individually or in combination (butyrate plus 5 or 10 mg/kg CF extract) with 3 cages per treatment and 3 animals per cage (*n* = 9), followed by daily challenges with *Clostridium perfringens* (CP) from d 14 to 17. Chickens were individually weighted on d 13 (**A**) and d 18 (**B**), and gross lesions of necrotic enteritis in the duodenum (**C**) and jejunum (**D**) were scored on d 18. The CP titers in the digesta of the jejunum (**E**) and cecum (**F**) were quantified using qPCR. Results were presented as means ± SEM (*n* = 9). Statistical significance (*P* < 0.05), denoted by uncommon superscripts, was determined using one-way ANOVA and post hoc Tukey’s test

To further evaluate the synergy between butyrate and FSK in the prevention of NE, microencapsulated sodium butyrate (1 g/kg) and FSK-containing CF extract (5 or 10 mg/kg) were supplemented to the basal diet individually or in combination 4 days prior to a 4-d challenge with *C. perfringens* to induce NE as described [19]. Chickens in all 6 treatments had a similar BW on d 13 prior to infection (*P* = 0.751; Fig. 2A), while *C. perfringens* challenge decreased weight gain by approximately 9% on d 18 as expected (Fig. 2B). Among *C. perfringens*-challenged groups, chickens fed 10 mg/kg CF extract alone had the lowest BW, while chickens administrated with a combination of sodium butyrate and 10 mg/kg CF extract had the highest BW on d 18 (Fig. 2B). While there was no lethality, *C. perfringens* infection caused characteristic lesions; however, the lesions in both the duodenum and jejunum were significantly reduced (*P* < 0.05) in chickens fed a combination of butyrate and 10 mg/kg CF extract, rather than either individually (Fig. 2C and D). Butyrate together with 5 mg/kg CF extract also slightly reduced the lesions in the duodenum (Fig. 2C) and jejunum (Fig. 2D).

*C. perfringens* colonization in the jejunum and cecum was also quantified among different groups using qPCR. Butyrate and CF extract alone as well as butyrate in combination with 5 mg/kg CF extract had a minimum impact on the *C. perfringens* titer, while butyrate and 10 mg/kg CF extract together reduced *C. perfringens* colonization by approximately 13-fold in the jejunum relative to the non-medication group (Fig. 2E). In the cecum, butyrate or FSK alone had a negligible effect on *C. perfringens* colonization, whereas a combination of butyrate with 5 or 10 mg/kg CF extract reduced *C. perfringens* numerically by approximately 3-fold (Fig. 2F). Overall, dietary supplementation of sodium butyrate with 10 mg/kg FSK-containing CF extract appeared to be synergistic in alleviating intestinal lesions and suppressing *C. perfringens* colonization in the intestinal tract of *C. perfringens*-challenged chickens.

### Regulation of HDP gene expression, barrier function, and inflammation in *C. perfringens*-challenged chickens by butyrate and FSK-containing plant extract

To understand the synergistic effect of butyrate and FSK on NE alleviation, the mRNA expressions of representative chicken HDP (*AvBD9* and *AvBD10*), barrier function (*MUC2*, *CLDN1*, and *TJP1*), and inflammatory cytokine (*IL-1β*) genes were measured in the jejunum of *C. perfringens-*challenged chickens on d 18. Relative to the non-infection control, 10 mg/kg CF extract alone or in combination with butyrate appeared to enhance the expressions of *AvBD9* (Supplementary Fig. S1A) and *AvBD10* (Supplementary Fig. S1B). The *C. perfringens* challenge increased *MUC2* expression, while CF extract alone or together with butyrate further seemed to improve *MUC2* expression, with a combination of butyrate and 10 mg/kg CF extract showing the most pronounced effect on *MUC2* induction (Supplementary Fig. S1C). *C. perfringens* infection reduced the expressions of *CLDN1* and *TJP1*, whereas the CF extract with or without butyrate tended to reverse the trend and enhanced both gene expressions (Supplementary Fig. S1D and S1E). Between 2 concentrations of the CF extract, butyrate combined with 10 mg/kg CF extract gave a better outcome in inducing *CLDN1* and *TJP1* expressions. Additionally, *IL-1β* gene expression was reduced in chickens fed a combination of butyrate and 10 mg/kg CF extract (Supplementary Fig. S1F). However, it is noted that none of the comparisons was statistically significant (*P* > 0.05). Nevertheless, dietary supplementation of butyrate with 10 mg/kg FSK-containing CF extract appeared to enhance the expressions of HDP and barrier function genes, while suppressing *IL-1β*, which is consistent with the protective effect of butyrate and FSK against subclinical NE.

### Supplementation of butyrate and FSK-containing plant extract on growth performance of broilers

To examine the effect of butyrate and FSK on the growth performance of healthy broilers, 2 feeding trials were conducted with Cobb chicks. In the first 21-d trial, dietary treatments had no effect on ADG throughout the entire trial (*P* > 0.05); however, 25 mg/kg CF extract or 1 g/kg microencapsulated butyrate in combination with 5, 10, or 25 mg/kg CF extract significantly reduced ADFI in the second week (*P* < 0.05), resulting in a significant improvement in FCR during the second week (*P* < 0.05) (Supplementary Table 1). Moreover, overall FCR between d 0–21 was not significantly different among treatments, although butyrate when combined with 10 or 25 mg/kg CF extract showed a numerical improvement as compared with the non-medicated control group (Supplementary Table 1).

**Fig. 3:**
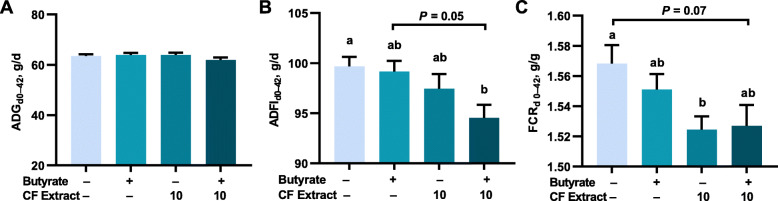
Influence of butyrate and forskolin (FSK) on growth performance of broilers. Day-of-hatch male Cobb chicks were supplemented with or without 1 g/kg microencapsulated sodium butyrate (diet), 10 mg/kg FSK-containing *Coleus forskohlii* (CF) extract individually or in combination with 9 replicate pens per treatment and 8 birds per pen (*n* = 9) for 42 d. Average daily gain (ADG), average daily feed intake (ADFI), and feed conversion ratio (FCR) were calculated for the entire period. Statistical significance was determined using one-way ANOVA, followed by post hoc Tukey’s test. The bars not sharing a common superscript are considered significantly different (*P* < 0.05)

A second feeding trial was further conducted for an entire growth cycle of 42 d to examine the influence of butyrate and FSK on growth performance of broilers. Again, ADG of Cobb chicks was largely unaffected by any dietary treatment (1 g/kg encapsulated butyrate, 10 mg/kg CF extract individually or in combination) during the 6-week trial (*P* > 0.05) (Fig. 3A), except for increased ADG in the second week in response to butyrate alone, relative to the combination (Table 2**)**. As compared to the control group, the butyrate/CF extract combination showed a significant reduction in ADFI between d 28–42 and also between d 0–42 (*P* < 0.05), while butyrate or CF extract alone had little impact (Table 2 and Fig. 3B). Overall feed efficiency between d 0–42 was significantly improved in chickens fed the butyrate/FSK combination (*P* < 0.05), relative to the control (Fig. 3B). Both feeding trials collectively suggested that a combination of butyrate and CF extract had no negative influence on the growth of broilers, but with a strong tendency to reduce feed intake and thus improve feed efficiency.

**Table 2:** Growth performance of broilers in a 42-d trial^1^

Items	Control^2^	Butyrate	FSK10	Butyrate +FSK10	SEM	*P*-value^3^
ADG, g/d						
d 0–7	15.8	16.2	15.8	15.4	0.31	0.38
d 7–14	40.8^ab^	42.4^a^	41.8^ab^	39.6^b^	0.55	0.01
d 14–28	80.6	82.2	80.2	80.8	1.10	0.63
d 28–42	104.7	102.4	106.3	98.9	2.27	0.19
d 0–42	63.6	63.9	63.9	61.9	0.81	0.27
ADFI, g/d						
d 0–7	23.0	25.2	22.7	24.0	1.08	0.22
d 7–14	60.9	59.7	57.1	57.9	1.21	0.13
d 14–28	114.5	117.6	115.6	113.7	1.55	0.33
d 28–42	182.1^a^	177.3^ab^	178.0^ab^	167.6^b^	3.17	0.02
d 0–42	99.7^a^	99.2^ab^	97.5^ab^	94.5^b^	1.20	0.02
FCR, g/g						
d 0–7	1.65	1.56	1.44	1.56	0.06	0.17
d 7–14	1.50^a^	1.41^ab^	1.37^b^	1.46^ab^	0.03	0.02
d 14–28	1.42	1.43	1.44	1.41	0.01	0.09
d 28–42	1.74	1.74	1.68	1.70	0.02	0.18
d 0–42	1.57^a^	1.55^ab^	1.52^b^	1.53^ab^	0.01	0.03

^1^The experiment was conducted with day-of-hatch male Cobb chicks with 9 replicate pens per treatment and 8 birds per pen (*n* = 9)

^2^Dietary treatments included: Control, basal diet; Butyrate, basal diet supplemented with 1 g/kg microencapsulated sodium butyrate; FSK10, basal diet supplemented with 10 mg/kg of 20% forskolin (FSK)-containing *Coleus forskohlii* (CF) extract; Butyrate+FSK10, basal diet supplemented with 1 g/kg microencapsulated sodium butyrate and 10 mg/kg CF extract

^3^ Statistical significance was determined using one-way ANOVA, followed by post hoc Tukey’s test. The values in a row not sharing a common superscript are considered significantly different (*P* < 0.05)

### Supplementation of butyrate and FSK-containing plant extract on carcass traits and meat quality of broilers

To ensure butyrate and FSK have no negative impact on carcass traits or meat quality, broilers were randomly selected from the second trial on d 28 and 48 and processed for the measurement of a series of carcass and meat quality traits. It was evident that butyrate and FSK individually or in combination had no significant effect on the yields of carcass and breast meat on either d 28 or d 42 (*P* > 0.05) (Table 3). It is noteworthy that abdominal fat was numerically reduced in d-28 chickens fed the butyrate/FSK combination, with further a significant decrease in d-42 chickens (*P* = 0.01). Such a reduction failed to be observed with butyrate or FSK alone. Butyrate and/or FSK had no significant effect on drop loss, an indicator of the water-holding capacity of the breast muscle on d 28 or d 42 (*P* > 0.05). The butyrate/FSK combination largely had no influence on either color or pH of the breast muscle on d 28 or d 42, except for a tendency to increase redness on d 28, relative to the non-medicated control group (Table 3). Overall, butyrate and FSK had little impact on either carcass traits or meat quality, except for a significant influence on reducing abdominal fat.

**Table 3:** Carcass traits and breast meat quality of broilers in a 42-d trial^1^

Items	Control^2^	Butyrate	FSK10	Butyrate +FSK10	SEM	*P*-value^3^
d 28						
Live BW, g	1420.3	1470.2	1468.3	1405.7	27.05	0.39
Carcass yield, %	62.72	62.89	63.03	63.53	0.67	0.88
Breast yield, %	28.39	27.92	27.78	27.44	0.43	0.50
Abdominal fat, %	1.79	1.74	1.82	1.69	0.09	0.79
Drip loss, %	0.59	0.98	0.88	0.73	0.12	0.13
Color lightness, L*	61.13	61.63	62.06	60.33	0.48	0.09
Color redness, a*	14.72^ab^	14.85^ab^	14.52^a^	15.32^b^	0.17	0.02
Color yellowness, b*	19.71	19.55	19.59	19.32	0.27	0.80
pH_24 h_	5.65^a^	5.61^ab^	5.55^b^	5.60^ab^	0.02	0.02
d 42						
Live BW, g	3074.0	3015.8	3098.0	3005.6	36.43	0.23
Carcass yield, %	70.67	71.17	71.53	70.45	0.57	0.54
Breast yield, %	21.72	21.78	22.48	22.16	0.18	0.43
Abdominal fat, %	1.25^a^	1.25^a^	1.24^a^	1.05^b^	0.05	0.01
Drip loss, %	1.90	2.61	1.96	1.82	0.37	0.51
Color lightness, L*	60.50	60.55	60.33	60.03	0.37	0.75
Color redness, a*	14.11	13.88	14.22	13.84	0.14	0.18
Color yellowness, b*	22.40	21.93	22.37	22.00	0.28	0.55
pH_24 h_	5.60	5.64	5.66	5.68	0.03	0.38

^1^The experiment was conducted with day-of-hatch male Cobb chicks with 9 replicate pens per treatment and 8 birds per pen. Three animals per pen (*n* = 27) and 5 animals per pen (*n* = 45) were sacrificed on d 28 on d 42, respectively, for subsequent analysis

^2^Dietary treatments included: Control, basal diet; Butyrate, basal diet supplemented with 1 g/kg microencapsulated sodium butyrate; FSK10, basal diet supplemented with 10 mg/kg of 20% forskolin (FSK)-containing *Coleus forskohlii* (CF) extract; Butyrate+FSK10, basal diet supplemented with 1 g/kg microencapsulated sodium butyrate and 10 mg/kg CF extract

^3^Statistical significance was determined using one-way ANOVA, followed by post hoc Tukey’s test. The values in a row not sharing a common superscript are considered significantly different (*P* < 0.05)

## Discussion

Antibiotic alternatives are needed in animal agriculture, given the restricted use of in-feed antibiotics in a growing number of countries [2, 3]. Host-directed strategies such as the induction of endogenous HDP synthesis are being actively explored against infectious diseases [3, 9, 10]. We previously showed that butyrate stimulates HDP synthesis and enhances the clearance of *Salmonella Enteritidis* in chickens [18] and that butyrate and FSK synergize with each other in inducing *AvBD9* gene expression both in vitro and in vivo [14]. In this study, we further demonstrated that dietary supplementation of butyrate and FSK synergizes to improve the expressions of *AvBD9* and *AvBD10* as well as barrier function genes such as *MUC2*, while suppressing inflammation in cell culture. Importantly, butyrate and FSK alleviates experimentally-induced NE in broilers in a synergistic manner. Furthermore, feeding butyrate and FSK has a strong tendency to improve feed efficiency and reduce abdominal fat with no negative impact on growth performance, carcass yield, or meat quality of broilers. Larger-scale animal trials are needed to confirm these promising results and provide a stronger statistical conclusion on some of the parameters measured in this study.

HDPs are critically important in animal immunity and disease resistance [4, 6]. Aberrant expression or deletion of HDP genes is often associated with increased susceptibility to infectious diseases [6, 8]. Downregulating HDP expression is specifically employed by certain bacteria to evade host innate immunity and establish infection [24]. In this study, *C. perfringens* infection also suppresses the expressions of *AvBD9* and *AvBD10* in the chicken jejunum; however, supplementation of butyrate and FSK reverses *C. perfringens*-mediated HDP suppression and, unsurprisingly, alleviates NE. Similarly, *C. perfringens* downregulates the expression of an antimicrobial peptide, *LEAP-2*, in the jejunum of chickens, and dietary supplementation of butyrate enhances *LEAP-2* expression and ameliorated NE [25]. These results collectively demonstrate that induction of HDPs represents a feasible approach to mitigate infectious diseases.

However, the mechanisms underlying butyrate- and FSK-mediated mitigation of NE appear to go beyond HDP induction. Mucins secreted by goblet cells and tight junctions connecting adjacent epithelial cells form important physiological and biochemical barriers between hosts and the external environment to maintain intestinal homeostasis [26, 27]. Butyrate is capable of maintaining intestinal barrier integrity by upregulating mucins and tight junction proteins [11], while we showed in this study that FSK enhances *MUC2* gene expression both in vitro and in vivo*.* Importantly, butyrate synergizes with FSK in promoting the transcription of *MUC2*, *CLDN1*, and *TJP1* in the intestinal tract.

Besides HDP induction and barrier function enhancement, butyrate/FSK-mediated protection of chickens from NE is also attributed in part to their synergy in suppressing inflammation, as evidenced in LPS-treated HD11 cells and *C. perfringens*-infected chicken jejunum. Consistent with well-known anti-inflammatory properties of butyrate [11, 28] and FSK [29, 30], we have shown that butyrate and FSK suppress *IL-1β* induction in LPS-stimulated HD11 cells and *C. perfringens*-infected jejunum individually and also in combination. Although the mechanism of action is not directly addressed in this study, the synergy in suppressing inflammatory response between butyrate and FSK is likely to act by inhibiting the activation of NF-κB and the NLRP3 inflammasome, 2 major targets for both butyrate and FSK [11, 28–30].

Consistent with earlier reports on mostly a negligible role of butyrate on growth performance of healthy animals [31–34], we also showed that dietary supplementation of 1 g/kg sodium butyrate has a minimum effect on weight gain, feed intake, or feed efficiency of broilers. However, FSK alone and particularly in combination with butyrate has a strong tendency to reduce feed intake and improve feed efficiency without affecting the growth rate of broilers in 2 feeding trials. Consistently, supplementation of FSK-containing CF extract has been shown to significantly reduce food intake and appetite in both rats [35] and humans [36]. This is perhaps unsurprising, given that FSK is a natural agonist of adenylyl cyclase and activates cAMP signaling [37, 38], which subsequently promotes lipolysis, thermogenesis, and loss of body fat without muscle mass loss [39–41], although the evidence on fat mass reduction is not entirely consistent [36]. Nevertheless, FSK is currently being explored to manage overweight and obesity in humans [42–44].

We observed that broilers fed a combination of butyrate and FSK show reduced abdominal fat deposition without affecting the carcass or breast muscle mass. However, FSK alone fails to reduce abdominal fat of broilers, perhaps due to the level of supplementation or a species difference. In rats, 50 g/kg of 1% CF extract was supplemented in the diet [35] and 2 mg/kg BW of FSK was administered to mice intraperitoneally every 2 days [40], while 250 mg of 20% CF extract was used in humans [36, 41]. In this study, we used 10 mg/kg of 20% CF extract in the diet. Apparently, optimal levels of dietary inclusion of FSK or FSK-containing CF extract remain to be investigated.

It is noted that, similar to FSK, butyrate is also known to reduce appetite and lipogenesis [12, 45], which perhaps helps explain a synergistic effect of butyrate and FSK in reducing feed intake and abdominal fat deposition, although butyrate or FSK alone at the levels used in this study fails to achieve such an effect. We used 1 g/kg sodium butyrate in this study. Sodium butyrate or butyric acid supplemented between 0.4 and 4 g/kg diet was reported earlier with no impact on carcass characteristics such as the carcass yield and carcass composition of broiler chickens [32, 46, 47]. However, dietary supplementation of 2 g/kg, but not 0.5 g/kg monobutyrin or a mixture of monobutyrin and tributyrin for 35 d decreased abdominal fat deposition and increased breast muscle composition of Ross 308 broilers [33]. Optimal concentrations of butyrate and its derivatives warrant further investigation for their effectiveness in regulating fat deposition.

## Conclusions

In summary, 2 mmol/L butyrate synergizes strongly with 5 μmol/L FSK in inducing the expressions of *AvBD9*, *AvBD10*, and *MUC2* in HD11 cells while suppressing LPS-induced *IL-1β* expression (*P* < 0.05). Dietary supplementation of 1 g/kg microencapsulated sodium butyrate and 10 mg/kg FSK-containing CF extract significantly reduces the lesions in both the duodenum and jejunum (*P* < 0.05), relative to non-medicated chickens. Furthermore, supplementation of 1 g/kg microencapsulated butyrate and 10 mg/kg CF extract for 42 d significantly reduced feed intake (*P* < 0.05) with a strong tendency to improve feed efficiency (*P* = 0.07) without affecting the growth rate of healthy broilers. Butyrate and FSK also synergistically decrease abdominal fat deposition (*P* = 0.01) without influencing the meat quality of broilers. Taken together, these results highlight a need to continue to explore the potential of combining butyrate with FSK as a promising antibiotic alternative to improve disease prevention, feed efficiency, and carcass composition in poultry and possibly other livestock species.

## Supplementary Information

**Additional file 1: Supplementary Material. Supplementary Table S1.** Growth performance of broilers in a 21-d trial. **Fig. S1.** Regulation of host defense peptide, barrier junction, and inflammatory cytokine gene expressions in the jejunum of *C. perfringens-*challenged chickens. Chickens were supplemented with or without microencapsulated sodium butyrate (1 g/kg diet), FSK-containing *Coleus forskohlii* (CF) extract (10 mg/kg) individually or in combination (butyrate plus 5 or 10 mg/kg CF extract) for 4 days prior to daily challenges with *C. perfringens* (CP) for 4 days. A segment of the mid-jejunum was collected from each animal on d 18 for gene expression analyses of *AvBD9* (**A**), *AvBD10* (**B**), *MUC2* (**C**), *CLDN1* (**D**), *TJP1* (**E**), and *IL-1β* (**F**) using RT-qPCR. Results were presented as means ± SEM (*n* = 9). Statistical significance was determined using one-way ANOVA and *post hoc* Tukey’s test.

## Data Availability

The datasets generated during the current study are included in this published article.

## Abbreviations

ADFI: Average daily feed intake
ADG: Average daily gain
AvBD: Avian β-defensin
cAMP: Cyclic adenosine monophosphate
CF: *Coleus forskohlii*
CLDN1: Claudin 1
FCR: Feed conversation ratio
MUC2: Mucin 2
HDP: Host defense peptide
FSK: Forskolin
GAPDH: Glyceraldehyde 3-phosphate dehydrogenase
IL-1β: Interleukin 1β
LPS: Lipopolysaccharide
NE: Necrotic enteritis
RT-qPCR: Quantitative reverse transcription PCR
SCFA: Short-chain fatty acid
SEM: Standard error of the mean
TJP1: Tight junction protein 1
