# Supplementary material for: Butyrate in combination with forskolin alleviates necrotic enteritis, increases feed efficiency, and improves carcass composition of broilers
Source: J Anim Sci Biotechnol. 2022 Feb 10;13:3. doi: 10.1186/s40104-021-00663-2 (PMC8830124; doi:10.1186/s40104-021-00663-2)
Supplement: Supplementary file 1 — Additional file 1: Supplementary Material. Supplementary Table S1. Growth performance of broilers in a 21-d trial. Fig. S1. Regulation of host defense peptide, barrier junction, and inflammatory cytokine gene expressions in the jejunum of C. perfringens-challenged chickens. Chickens were supplemented with or without microencapsulated sodium butyrate (1 g/kg diet), FSK-containing Coleus forskohlii (CF) extract (10 mg/kg) individually or in combination (butyrate plus 5 or 10 mg/kg CF extract) for 4 days prior to daily challenges with C. perfringens (CP) for 4 days. A segment of the mid-jejunum was collected from each animal on d 18 for gene expression analyses of AvBD9 (A), AvBD10 (B), MUC2 (C), CLDN1 (D), TJP1 (E), and IL-1β (F) using RT-qPCR. Results were presented as means ± SEM (n = 9). Statistical significance was determined using one-way ANOVA and post hoc Tukey’s test. [file 40104_2021_663_MOESM1_ESM.docx]

**Supplementary Material**

**Supplementary Table S1.** Growth performance of broilers in a 21-d trial^1^

| Items | Control^2^ | Butyrate | FSK25 | Butyrate  +FSK5 | Butyrate  +FSK10 | Butyrate  +FSK25 | SEM | *P*-value^3^ |
| --- | --- | --- | --- | --- | --- | --- | --- | --- |

| ADG, g/d |
| --- |

| d 0–7 | 14.8 | 15.3 | 15.1 | 15.2 | 15.0 | 15.2 | 0.09 | 0.61 |
| --- | --- | --- | --- | --- | --- | --- | --- | --- |
| d 7–14 | 31.6 | 33.0 | 33.2 | 32.1 | 31.8 | 33.3 | 0.34 | 0.53 |
| d 14–21 | 56.8 | 56.1 | 55.4 | 54.9 | 55.8 | 56.7 | 0.36 | 0.65 |
| d 0–21 | 34.4 | 35.0 | 34.6 | 34.5 | 34.5 | 35.0 | 0.16 | 0.78 |

| ADFI, g/d |
| --- |

| d 0–7 | 19.3 | 20.2 | 19.0 | 18.6 | 18.5 | 18.8 | 0.21 | 0.20 |
| --- | --- | --- | --- | --- | --- | --- | --- | --- |
| d 7–14 | 51.8^a^ | 50.5^ab^ | 48.3^b^ | 47.1^b^ | 47.2^b^ | 48.5^ab^ | 0.51 | 0.03 |
| d 14–21 | 88.5 | 90.6 | 90.0 | 90.9 | 89.5 | 89.9 | 0.58 | 0.90 |
| d 0–21 | 52.6 | 53.3 | 52.8 | 52.1 | 51.4 | 51.8 | 0.30 | 0.55 |

| FCR, g/g |
| --- |

| d 0–7 | 1.30 | 1.32 | 1.26 | 1.22 | 1.23 | 1.24 | 0.01 | 0.07 |
| --- | --- | --- | --- | --- | --- | --- | --- | --- |
| d 7–14 | 1.65^a^ | 1.53^ab^ | 1.46^b^ | 1.47^b^ | 1.49^b^ | 1.46^b^ | 0.02 | <0.01 |
| d 14–21 | 1.56^b^ | 1.62^ab^ | 1.62^ab^ | 1.66^a^ | 1.60^b^ | 1.59^b^ | 0.01 | 0.03 |
| d 0–21 | 1.53 | 1.52 | 1.50 | 1.51 | 1.49 | 1.48 | 0.01 | 0.10 |

^1^ The experiment was conducted for 21-days with day-of-hatch male Cobb chicks with 6 replicate pens per treatment and 8 birds per pen (*n* = 6).

^2^ Dietary treatments included: Control, basal diet; Butyrate, basal diet supplemented with 1 g/kg microencapsulated sodium butyrate; FSK25, the basal diet supplemented with 25 mg/kg of 20% forskolin-containing *Coleus forskohlii* (CF) extract; Butyrate+FSK5/FSK10/FSK25, basal diet supplemented with 1 g/kg microencapsulated sodium butyrate and 5, 10, or 25 mg/kg CF extract, respectively.

^3^ Statistical significance was determined using one-way ANOVA, followed by *post hoc* Tukey’s test. The values in a row not sharing a common superscript are considered significantly different (*P* < 0.05).

**Fig. S1.** Regulation of host defense peptide, barrier junction, and inflammatory cytokine gene expressions in the jejunum of C. perfringens-challenged chickens. Chickens were supplemented with or without microencapsulated sodium butyrate (1 g/kg diet), FSK-containing Coleus forskohlii (CF) extract (10 mg/kg) individually or in combination (butyrate plus 5 or 10 mg/kg CF extract) for 4 days prior to daily challenges with C. perfringens (CP) for 4 days. A segment of the mid-jejunum was collected from each animal on d 18 for gene expression analyses of AvBD9 (A), AvBD10 (B), MUC2 (C), CLDN1 (D), TJP1 (E), and IL-1β (F) using RT-qPCR. Results were presented as means ± SEM (n = 9). Statistical significance was determined using one-way ANOVA and post hoc Tukey’s test.
